# Digital Health in Early Childhood: A Cross-Sectional Study of Pediatricians’ Knowledge, Practices, and Training Needs in Northern Italy

**DOI:** 10.3390/healthcare13222945

**Published:** 2025-11-17

**Authors:** Viola Trevisani, Isotta Zinani, Silvia Cattani, Elena Ferrari, Lorenzo Iughetti, Laura Lucaccioni

**Affiliations:** 1 Pediatric Unit, Department of Medical and Surgical Sciences of the Mother, Children and Adults, University of Modena and Reggio Emilia, 41125 Modena, Italy; 2 PhD Program in Clinical and Experimental Medicine, University of Modena and Reggio Emilia, 41125 Modena, Italy; 3 Post-Graduate School of Pediatrics, University of Modena and Reggio Emilia, 41125 Modena, Italy; 4 Primary Care Pediatrics, AUSL of Modena, 41122 Modena, Italy; 5 Primary Care Pediatrics, AUSL of Reggio Emilia, 42122 Reggio Emilia, Italy

**Keywords:** digital devices, early childhood, pediatricians, digital education, health promotion

## Abstract

**Background:** Digital devices (DDs) are increasingly present in early childhood, with screen exposure beginning as early as infancy. Despite international and national guidelines discouraging digital media use before age two, many children are exposed to screens far earlier, often mediated by parents and caregivers. Excessive or unregulated screen use has been linked to adverse neurodevelopmental, emotional, and physical outcomes. **Objective:** This study aims to assess the knowledge, attitudes, and educational needs of primary care pediatricians (PCPs) regarding digital education (DE) and DD use in preschool-aged children (0–6 years) in two provinces of Northern Italy. **Methods:** A cross-sectional survey was distributed to all 165 PCPs in the Modena and Reggio Emilia provinces between December 2024 and January 2025. The 17-item questionnaire explored PCPs’ knowledge of guidelines, awareness of DD-related risks, current counseling practices, and training needs. **Results:** Of the 165 contacted PCPs, 93 (56%) completed the survey. While 77% were aware of Italian Pediatric Society recommendations, only 56% correctly identified age two as the threshold for total screen avoidance. 87% of PCPs recognized the risks of excessive DD use, particularly its cognitive, behavioral, and physical consequences. Nearly all participants (95%) reported discussing DE during clinical visits, and 96% expressed a desire for further training. **Conclusions:** PCPs show strong engagement in promoting healthy digital habits but lack specific knowledge of current recommendations. Structured tools such as digital health check-ups and targeted training programs are needed to strengthen pediatricians’ roles in digital health education and support parental guidance.

## 1. Introduction

Digital screens have become an integral component of the early childhood environment, with exposure beginning as early as infancy. According to a 2022 report by the Italian National Institute of Health (Istituto Superiore di Sanità—ISS) [1], 22.1% of children aged 2 to 5 months in Italy are already exposed to screens, and this percentage increases with age. In particular, in the Emilia-Romagna region, 56.5% of children were reported to spend time on screens already at 12 months postpartum.

Parents, as primary users of digital devices (DDs), often represent the first point of contact through which young children are introduced to screen-based technologies [2,3]. In many households, screens are now routinely used for entertainment, communication, and even as tools for calming or distracting children during daily routines.

A growing body of evidence underscores the potentially harmful effects of both insufficiently regulated and excessive DDs use during early developmental periods [4,5,6,7]. Early and prolonged exposure to screens has been associated with negative outcomes in neurocognitive development, learning processes, and general well-being [8]. These impacts are particularly concerning during the first six years of life, a critical window for brain development, language acquisition, emotional regulation, and social skills formation [9]. Furthermore, excessive screen use has been linked to delays in achieving developmental milestones, such as language development and attention regulation [10,11].

Notably, the nature of screen interaction also plays a crucial role. Passive screen consumption—such as watching videos without adult interaction—has been shown to have more detrimental effects compared to interactive or educational content that is co-viewed and discussed with a caregiver [12]. The quality of content, context of use, and parental mediation are therefore essential factors in determining the developmental impact of digital media.

Beyond cognitive and emotional domains, prolonged screen exposure has also been associated with disruptions in physical development. Studies have identified negative effects on visual and auditory systems, including increased risk of digital eye strain and reduced auditory processing due to overstimulation or lack of verbal interaction [13]. Additionally, sedentary behaviors associated with extended screen time contribute to metabolic dysregulation, increased risk of childhood obesity, and emerging concerns regarding cardiovascular health [4,14].

Over the past decade, children’s engagement with digital devices has steadily increased, with screen exposure occurring at progressively younger ages. This trend is paralleled by documented repercussions on both neurocognitive and psychophysical development, prompting national and international health authorities—such as the World Health Organization (WHO) [15], the American Academy of Pediatrics (AAP) [4], and the Italian Pediatric Society (SIP) [16]—to issue specific guidelines on appropriate screen time and digital device use in early childhood.

Despite the dissemination of these guidelines, a substantial gap in awareness persists among both caregivers and healthcare professionals regarding the potential risks associated with inappropriate digital device use. This lack of awareness may hinder the adoption of effective preventive strategies and limit the provision of developmentally informed parental guidance. As a result, digital health literacy and education have become pressing public health priorities, particularly in pediatric settings.

Primary care pediatricians (PCPs) play a central role in addressing these challenges. As frontline healthcare providers, they are uniquely positioned to offer anticipatory guidance to families, support informed decision-making, and promote the safe and developmentally appropriate use of technology. Their responsibilities extend beyond risk mitigation, encompassing the facilitation of positive digital experiences that align with children’s developmental needs.

The aim of this study is to assess the knowledge of PCPs regarding the use of DDs in preschool-aged children (0–6 years). Specifically, the study evaluates PCPs’ awareness of the potential effects of digital exposure on neurocognitive development and overall well-being, as well as their perceived role in digital education. Furthermore, the research seeks to identify existing gaps in PCPs’ understanding, examine their competencies in managing the risks and benefits associated with digital media, and propose targeted strategies to improve professional training and parental counseling in this area.

To investigate these objectives, a cross-sectional study was conducted across two neighboring provinces in Northern Italy—Modena and Reggio Emilia. The study involved the distribution of a structured survey to all PCPs practicing in these areas.

## 2. Materials and Methods

A digital, self-administered questionnaire consisting of 17 items was developed to assess the knowledge, attitudes, and concerns of PCPs regarding digital education (DE) and the use of DDs in early childhood. The survey was specifically designed for PCPs practicing in the provinces of Modena and Reggio Emilia, located in the Emilia-Romagna region of Northern Italy.

The questionnaire was distributed via email to all 165 PCPs working in these areas between December 2024 and January 2025. Participation was entirely voluntary and anonymous. In December 2024, participants received an email invitation containing a brief explanation of the study objectives and a direct link to the survey, which was administered through Google Forms. To ensure data completeness and quality, most questions were marked as mandatory; respondents could not submit the form unless those items were completed. The data collection period concluded at the end of January 2025.

The survey included a total of 17 questions: 3 open-ended items and 14 multiple-choice questions, of which 11 required mandatory responses. The estimated time for completion was approximately 5 min. Duplicate submissions were prevented by restricting responses to one per participant.

The questionnaire was developed based on current literature and the recommendations of the Italian Society of Pediatrics (SIP) and the American Academy of Pediatrics (AAP). To ensure content validity, it was reviewed by three senior pediatricians. Before distribution, a pilot test was conducted with a small group of five primary care pediatricians to evaluate clarity, comprehension, and completion time, and minor wording adjustments were incorporated based on their feedback.

The questionnaire was structured to gather:

- Demographic and professional information (e.g., number of patients managed, district of practice),
- Basic knowledge of digital education and the use of digital devices in early childhood,
- Familiarity with national guidelines, specifically those issued by the SIP,
- The degree to which these guidelines are incorporated into routine clinical practice, and
- PCPs’ perceived need for additional training in this area.

The full questionnaire and response options are provided in Appendix A.

### 2.1. Statistical Analysis

All data were analyzed using Statistical Package for the Social Sciences (SPSS) software, version 20.0 (IBM SPSS Inc., Chicago, IL, USA). Responses to multiple-choice questions were summarized using frequencies and percentages. For each item, percentages were calculated based on the number of respondents who answered the specific question. Open-ended questions were analyzed based on the nature of the response: quantitative responses (e.g., numerical values such as number of patients) were summarized using mean ± standard deviation (SD), and qualitative responses (e.g., free-text comments and opinions) were analyzed using a basic thematic analysis. Responses were grouped according to recurring themes or categories, and representative examples were identified to illustrate common viewpoints.

### 2.2. Ethical Considerations, Patient Information, and Written Informed Consent

This study did not require the approval of an ethics committee because the questionnaire data were anonymous, making it impossible to identify and harm any respondent. Moreover, neither drugs nor medical devices were prescribed or administered. As a result, the responses were collectively examined while taking into account Italian and European regulations governing the management of personal data [17,18,19].

In more detail, anonymity was granted by not requesting the name, surname, and date of birth. In order to retrieve information about the geographical area of participants while still preserving anonymity, the province in which the participants lived was asked instead of their address. To further protect identities, it was possible to choose not to specify a gender.

The cover letter of the questionnaire informed the participants that the data would be used only for scientific purposes, that the raw data would be archived for a maximum of five years, and they would be accessible only by researchers at the University of Modena and Reggio Emilia. Google Forms was used only to spread the questionnaire and to collect data. All the collected data were then downloaded and stored on a professional computer and protected by a password.

It was possible to complete the questionnaire only after the participants declared that they understood the methods and purposes of the study by clicking on the “I give the consent” option in reference to the processing of personal data. Participants who disagreed were redirected to a thank you message.

## 3. Results

The total number of PCPs in the provinces of Modena and Reggio Emilia is 165, with 94 practicing in the Modena area and 71 in the Reggio Emilia area.

A total of 93 PCPs (56% of the PCPs contacted), fully completed the survey within the designated timeframe. The response rate of 56% was comparable between the two provinces with 52 out of 94 PCPs responding in Modena (55.3%) and 41 out of 71 PCPs responding in Reggio Emilia (57.7%).

Among the responders, 73% practiced in urban district. The average number of patients per PCP was 801 ± 134 (SD), of whom on average of 340 ± 88 (SD) were children aged 0–6 years.

### 3.1. Knowledge About the Recommendations on the Correct Use of DDs Stated by the Italian Pediatric Society (SIP)

**Figure 1:**
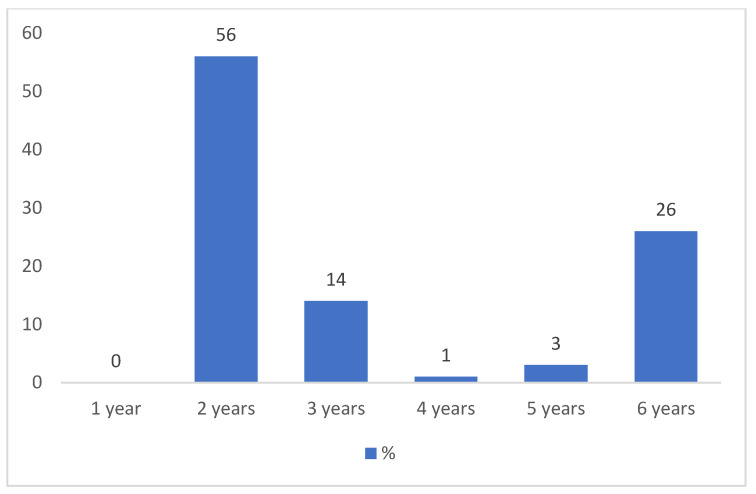
Percentage distribution of PCPs’ responses to the question: “According to your knowledge, up to what age should the use of digital devices be completely avoided?” (question no. 7).

Although most PCPs (77%) reported awareness of the SIP recommendations on the appropriate use of DDs, only 56% correctly identified the recommended age—up to 2 years—as the threshold for complete avoidance of DDs. A notable proportion (26%) recommended avoiding DDs until 6 years of age. Other responses included 3 years (14%), 5 years (3%), and 4 years (1%). Importantly, none of the respondents indicated 1 year as the appropriate maximum age for total avoidance of DDs, suggesting that all participants are aware that screen exposure should be completely avoided during the first year of life (Figure 1).

**Figure 2:**
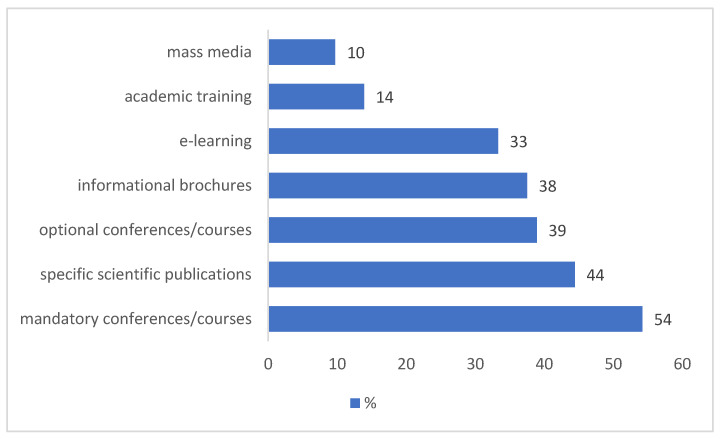
Channels through which PCPs reported obtaining information on SIP recommendations (*n* = 72). Multiple answers were allowed (question no. 6).

For the 72 respondents who confirmed awareness of SIP guidelines, the primary sources of information regarding SIP recommendations were mandatory meetings (39 answers, 54%) and scientific literature (32 answers, 44%). Less frequently, PCPs reported acquiring information knowledge through online distance learning (24 answers, 33%), information brochures (27 answers, 37%), academic training (10 answers, 14%), and information through the media (7 answers, 10%) (Figure 2).

### 3.2. Knowledge of the Adverse Effects of Excessive DDs Use

**Figure 3:**
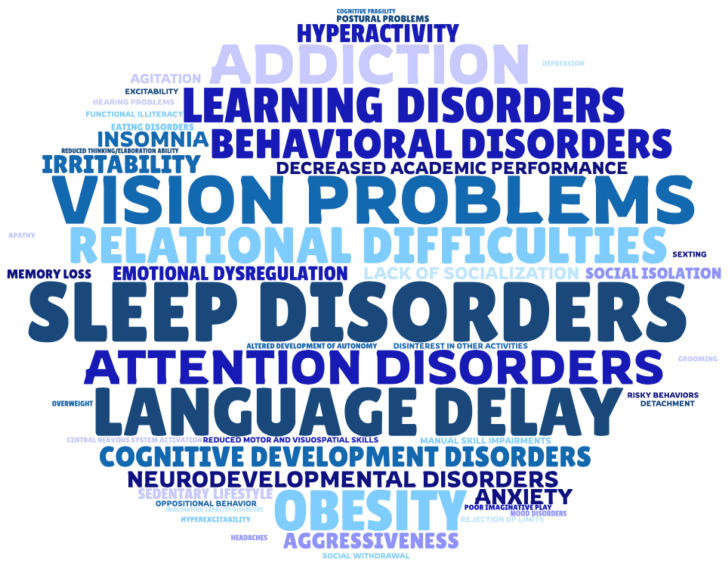
Wordcloud illustrating the range and frequency of long-term consequences associated with excessive digital device use in preschool children, as reported by primary care providers (PCPs). The size of each word reflects the relative frequency with which each consequence was mentioned, highlighting the most commonly recognized effects. Detailed data underlying the word cloud are provided in the Supplementary Table S1.

A total of 87% of PCPs reported being aware of the long-term adverse effects associated with excessive exposure to DDs in preschool-aged children. The reported consequences, summarized in Figure 3, can be categorized into six main domains: cognitive and neurodevelopmental effects, emotional and behavioral issues, physical health impacts, developmental and motor issues, social and relational difficulties, and digital risk behaviors.

Furthermore, the majority of respondents accurately identified specific contexts in which DD use should be avoided, including during meals, within one hour prior to bedtime, and as a means of emotional regulation (e.g., used as a pacifier).

### 3.3. PCPs’ Practice in Terms of Digital Education and Their Knowledge Demands

There was a strong consensus among PCPs on the importance of educating parents about the impacts of DDs, with 98% emphasizing the need for parental guidance in this area. The vast majority of PCPs (95%) reported routinely addressing DE during health check-ups, offering practical advice to help parents limit their children’s use of DDs. Additionally, 94% considered the distribution of brief informational brochures on DE within clinical practice to be both useful and beneficial.

**Figure 4:**
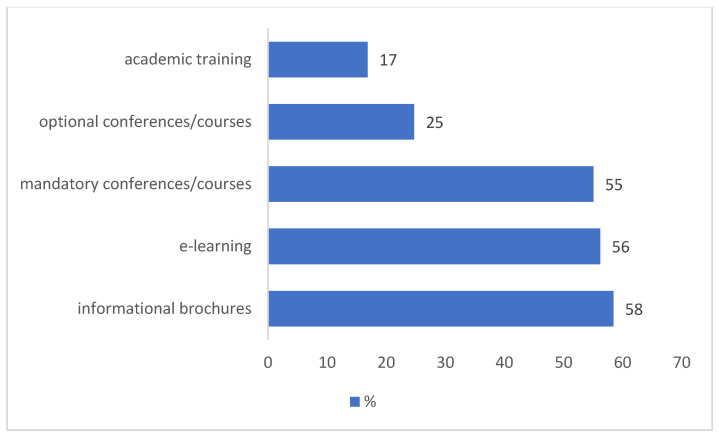
Channels through which PCPs would like to obtain more information on digital education (*n* = 89). Multiple answers were allowed (question no. 16).

Despite this, 96% of PCPs expressed a need for further training in digital health. Of the 89 respondents who desired more information, the preferred educational formats included mandatory face-to-face learning (49 preferences, 55%), distance learning (50 preferences, 50%), and informational brochures (52 preferences, 58%). A smaller proportion (15 preferences, 17%) indicated a preference for academic training as a means of enhancing their knowledge on the subject (Figure 4).

## 4. Discussion

To our knowledge, this is one of the first study in Italy to investigate PCPs knowledge, attitudes, and educational needs concerning DE and the use of DDs in early childhood. The results from PCPs in the provinces of Modena and Reggio Emilia offer a valuable snapshot of current practices, levels of awareness, and existing training gaps within this key professional group.

Our findings reveal both encouraging engagement and notable areas for improvement. On one hand, PCPs demonstrate a strong and proactive commitment to addressing digital health topics in daily clinical practice. The widespread recognition of the usefulness of informational tools—such as brochures—highlights a practical and family-centered approach to parental education. This not only reflects awareness of the topic’s relevance but also a concrete willingness to adopt strategies that support behavioral change within families. These findings are consistent with the growing international literature that underscores the central role of pediatricians in promoting healthy digital habits [20,21].

On the other hand, a significant knowledge gap persists. While many participants reported general familiarity with the recommendations issued by the SIP, only a portion correctly identified key elements—such as the recommendation to avoid any digital device use before the age of two. Similar gaps between awareness and detailed knowledge have been documented elsewhere, including in a Canadian study in which pediatricians were aware of screen time guidelines but struggled with their clinical implementation [21].

In light of these findings, the concept of digital check-ups emerges as a promising strategy to systematize and strengthen pediatricians’ role in digital health promotion. Given the trusted and continuous relationship between families and PCPs, the pediatric setting represents an ideal environment for implementing behavioral interventions related to digital exposure. Integrating digital health check-ups into routine well-child visits may offer several key benefits, including enhancing parental awareness of the long-term risks associated with early and excessive screen exposure [4]; delivering targeted guidance to foster healthy digital habits within the family; enabling the early identification of behavioral or developmental concerns potentially linked to problematic screen use [22]; and strengthening communication between pediatricians and caregivers around the challenges of digital parenting. This approach aligns with the broader framework of anticipatory guidance in pediatrics, placing digital health alongside other critical domains of early childhood development such as nutrition, sleep, and emotional regulation [4].

Supporting this approach, the Digital Guardians program conducted in Friuli-Venezia Giulia (Northern Italy) provides compelling evidence of the effectiveness of integrating digital health check-ups into routine pediatric care [23]. This quasi-experimental study involved over 1000 families and provided tailored guidance on screen media use through family pediatricians during standard well-child visits. Remarkably, after only three months, families who received the intervention demonstrated statistically significant improvements in both managing their children’s digital device use and their confidence in engaging in alternative, non-digital interactions. These findings highlight the potential of structured pediatric counseling as a vital component of early preventive care in the digital age.

Given the specific needs identified in our region, expanding the implementation of such digital health check-ups is likely to yield substantial benefits for the health and well-being of young children. Broader adoption of structured, pediatrician-led digital counseling could play a crucial role in promoting healthier developmental trajectories and supporting families as they navigate the complexities of raising children in an increasingly digital world.

This study represents a meaningful contribution to the understanding of digital education within community-based pediatric care. Its strengths include a respectable response rate (56%) and good territorial representation, including both urban and semi-urban settings. Nonetheless, certain limitations must be acknowledged. The potential influence of social desirability bias may have led to an overestimation of PCPs’ actual engagement in digital education practices. In addition, due to the small and closely connected sample, demographic data such as age or gender were not collected to prevent possible identification of participants. This decision, while essential to ensure confidentiality, limited the possibility of exploring associations with these variables. Despite these limitations, the study provides valuable insights and a sound basis for future, larger-scale research on digital education in pediatric primary care.

## 5. Conclusions

In conclusion, PCPs in the provinces of Modena and Reggio Emilia demonstrate a strong commitment to promoting digital education within families. However, significant gaps remain in their knowledge of current recommendations, underlining the need for targeted educational support. The development of structured tools—such as digital check-ups—and the provision of comprehensive and accessible training programs represent crucial steps toward reinforcing the pediatrician’s role in this evolving domain. In today’s highly connected world, the pediatrician’s guidance in digital health is not only timely and relevant, but essential for safeguarding child development and supporting families in navigating the digital environment.

## Data Availability

The database used and analyzed during the current study is available from the corresponding author on reasonable request due to the ongoing nature of the research project.

## Supplementary Materials

The following supporting information can be downloaded at: https://www.mdpi.com/article/10.3390/healthcare13222945/s1, Table S1: Long-term consequences of excessive digital device use in preschool children, as reported by primary care providers (PCPs). For each consequence, the table shows the number and percentage of PCPs who mentioned it, providing the quantitative details underlying Figure 3.

## Abbreviations

The following abbreviations are used in this manuscript:

DD	Digital devices
DE	Digital Education
PCPs	Primary Care Pediatricians
SD	Standard Deviation
SIP	Italian Pediatric Society

## Appendix A

This appendix includes the full version of the questionnaire used in the study, presented in both the original Italian and its English translation.

*Italian version:*

1. Quanti sono i suoi assistiti in totale? (specificare il numero)
  → _____
2. Quanti sono i suoi assistiti sotto i 6 anni? (specificare il numero)?
  → _____
3. In che provincia lavora?
  □ Modena
  □ Reggio Emilia
4. Il distretto in cui lavora, si può definire:
  □ Zona urbana o zona industrializzata
  □ Zona rurale
5. Conosce le raccomandazioni SIP sull’utilizzo dei Dispositivi Digitali? *
  □ Si
  □ No
6. Se SI, tramite quali canali ha avuto informazioni su tale argomento: (possibili più scelte) Seleziona tutte le voci applicabili.
  □ Formazione accademica
  □ Congressi/Corsi obbligatori
  □ Congressi/Corsi facoltativi FAD
  □ Opuscoli informativi
  □ Pubblicazioni scientifiche specifiche
  □ Mass media
7. Sa fino a che età (anni) sarebbe opportuno evitare completamente l’utilizzo dei Dispositivi Digitali?
  □ 1
  □ 2
  □ 3
  □ 4
  □ 5
  □ 6
8. Conosce i limiti di esposizione ai Dispositivi Digitali a seconda delle fasce di età?
  □ Si
  □ No
9. In quale delle seguenti occasioni sarebbe opportuno evitare l’utilizzo dei Dispositivi Digitali? (possibili più scelte). Seleziona tutte le voci applicabili.
  □ Durante i pasti
  □ Almeno 1 ora prima di andare a letto
  □ In caso di programmi a ritmo sostenuto, applicazioni con contenuti distraenti o violenti
  □ Per tenere i bambini tranquilli in luoghi pubblici
  □ Durante le visite mediche
10. Conosce gli effetti a lungo termine dell’esposizione ai Dispositivi Digitali in età prescolare?
  □ Si
  □ No
11. Se SI, può elencarli? (almeno 3)
  → _______________________________________
12. Durante i bilanci di salute affronta tale argomento con i genitori dei suoi assistiti?
  □ Si
  □ No
13. Se SI, fornisce consigli pratici ai genitori, tentando quindi di limitare l’esposizione a Dispositivi Digitali dei suoi assistiti?
  □ Si
  □ No
14. Quanto ritiene importante informare i genitori dei suoi assistiti sugli effetti dei Dispositivi Digitali?
  □ Non importante
  □ Importante ma non fondamentale
  □ Molto importante
15. Vorrebbe più informazioni sull’argomento?
  □ Si
  □ No
16. Se SI, quale tipo di formazione preferirebbe? (possibili più scelte). Seleziona tutte le voci applicabili.
  □ Formazione accademica
  □ Congressi/Corsi obbligatori
  □ Congressi/Corsi facoltativi
  □ FAD
  □ Opuscoli informativi
17. Ritiene utile la diffusione di brevi brochures su tale argomento nella sua pratica clinica (e.g., opuscoli specifici da lasciare in sala d’attesa)?
  □ Si
  □ No
  □ Non so

*English version:*

1. What is your total patient load? (Please specify the number)
  → _____
2. How many children under the age of 6 are currently in your care? (Please specify the number)
  → _____
3. In which province do you practice?
  □ Modena
  □ Reggio Emilia
4. How would you classify the area in which you practice?
  □ Urban or industrialised area
  □ Rural area
5. Are you familiar with the SIP (Italian Pediatric Society) recommendations on the use of digital devices in early childhood?
  □ Yes
  □ No
6. If yes, through which channels did you receive this information? (Multiple answers allowed)
  □ Academic training
  □ Mandatory congresses/courses
  □ Optional congresses/FAD (distance learning)
  □ Informational brochures
  □ Scientific publications
  □ Mass media
7. According to your knowledge, up to what age (years) should the use of digital devices be completely avoided?
  □ 1
  □ 2
  □ 3
  □ 4
  □ 5
  □ 6
8. Are you aware of the recommended screen time limits for different age groups?
  □ Yes
  □ No
9. In which of the following situations should digital device use be avoided? (Multiple answers allowed)
  □ During meals
  □ At least one hour before bedtime
  □ During exposure to fast-paced, distracting, or violent content
  □ To calm or distract children in public places
  □ During medical consultations
10. Are you aware of the potential long-term effects of digital device use in preschool-aged children?
  □ Yes
  □ No
11. If yes, please list at least three of these long-term effects:
  → _______________________________________
12. Do you address the topic of digital device use with parents during routine health visits?
  □ Yes
  □ No
13. If yes, do you provide practical advice to help parents limit their children’s screen time?
  □ Yes
  □ No
14. How important do you think it is to inform parents about the effects of digital device use in early childhood?
  □ Not important
  □ Important but not essential
  □ Very important
15. Would you like to receive more information or training on this topic?
  □ Yes
  □ No
16. If yes, what type of training would you prefer? (Multiple answers allowed)
  □ Academic training
  □ Mandatory congresses/courses
  □ Optional congresses/courses
  □ FAD (distance learning)
  □ Informational brochures
17. Do you think it would be useful to distribute short brochures on this topic in your clinical practice (e.g., brochures available in the waiting room)?
  □ Yes
  □ No
  □ Not
