# Supplementary material for: Digital Health in Early Childhood: A Cross-Sectional Study of Pediatricians’ Knowledge, Practices, and Training Needs in Northern Italy
Source: Healthcare (Basel). 2025 Nov 17;13(22):2945. doi: 10.3390/healthcare13222945 (PMC12652123; doi:10.3390/healthcare13222945)
Supplement: Supplementary file 1 [file healthcare-13-02945-s001.zip › healthcare-3967569-supplementary.pdf]

healthcare

MDPI

Article

Supplementary

Table S1 - Long-term consequences of excessive digital device use in preschool children, as reported by primary care providers (PCPs). For each consequence, the table shows the number and percentage of PCPs who mentioned it, providing the quantitative details underlying Figure 3.

Long-term consequences (english)	Long-term consequences (italian)	n
Sleep disorders	Disturbi del sonno	42
Attention disorders	Disturbi dell'attenzione	29
Vision problems	Problemi di vista	21
Language delay	Ritardo del linguaggio	16
Neurodevelopment disorders	Disturbi del neurosviluppo	16
Relational difficulties	Disturbi relazionali	16
Obesity	Obesità	15
Cognitive impairment disorders	Deficit di concentrazione	14
Learning disorders	Disturbi dell'apprendimento	14
Addiction	Dipendenza	13
Behavioral disorders	Disturbi comportamentali	13
Hyperactivity	Iperattività	9
Aggressivness	Aggressività	7
Emotional dysregulation	Disregolazione emotiva	7
Irritability	Irritabilità	6
Social isolation	Isolamento	6
Anxiety	Ansia	5
Insomnia	Insonnia	5
Agitation	Agitazione	4
Decreased academic performance	Ridotto rendimento scolastico	4
Lack of socialization	Mancata socializzazione	4
Sedentary lifestyle	Sedentarietà	4
Eating disorders	Disturbi dell'alimentazione	3
Memory loss	Calo della memoria	3
Postural problems	Problemi posturali	3
Headaches	Cefalea	2
Manual skills impairment	Alterazioni della manualità	2
Mood disorders	Disturbi dell'umore	2
Overweight	Sovrappeso	2
Altered development of autonomy	Alterazione dello sviluppo dell'autonomia	1
Apathy	Apatia	1
Central nervous system activation	Attivazione del SNC	1
Cognitive fragility	Fragilità cognitiva	1
Depression	Depressione	1
Detachment	Estraniamento	1
Disinteress in other activities	Disinteresse per altre attività	1
Excitability	Eccitabilità	1
Functional illiteracy	Analfabetismo funzionale	1
Grooming	Grooming	1
Hearing problems	Problemi di udito	1
Hyperexcitability	Ipereccitabilità	1
Immagination capacity disorders	Disturbi della capacità immaginativa	1

 https://doi.org/10.3390/healthcare13222945

Oppositional behavior	Comportamenti oppositivi	1
Poor immaginative play	Scarsi giochi di fantasia	1
Reduced motor and visuospatial skills	Minori abilità motorie e visuo-spaziali	1
Reduced thinking/elaboration ability	Minore capacità elaborativa di pensiero	1
Rejection of limits	Rifiuto dei limiti	1
Risky behaviors	Comportamenti a rischio	1
Sexting	Sexting	1
Social withdrawl	Minore interazione con i coetanei	1
